# Poly(dl-lactide) Polymer Blended with Mineral Phases for Extrusion 3D Printing—Studies on Degradation and Biocompatibility

**DOI:** 10.3390/polym16091254

**Published:** 2024-04-30

**Authors:** Corina Vater, Christian Bräuer, Stefanie Grom, Tatjana Fecht, Tilman Ahlfeld, Max von Witzleben, Anna-Maria Placht, Kathleen Schütz, Jan Marc Schehl, Tobias Wolfram, Frank Reinauer, Martin Scharffenberg, Jakob Wittenstein, Andreas Hoess, Sascha Heinemann, Michael Gelinsky, Günter Lauer, Anja Lode

**Affiliations:** 1 Centre for Translational Bone, Joint, and Soft Tissue Research, University Hospital Carl Gustav Carus and Faculty of Medicine at Technische Universität Dresden, 01307 Dresden, Germany; tilman.ahlfeld@tu-dresden.de (T.A.); max.von_witzleben@tu-dresden.de (M.v.W.); anna-maria.placht@tu-dresden.de (A.-M.P.); kathleen.schuetz@tu-dresden.de (K.S.); michael.gelinsky@tu-dresden.de (M.G.); 2 Department of Oral and Maxillofacial Surgery, University Hospital Carl Gustav Carus at Technische Universität Dresden, 01307 Dresden, Germany; christian.braeuer@med.uni-rostock.de (C.B.); guenter.lauer@uniklinikum-dresden.de (G.L.); 3 KLS Martin SE & Co. KG, 78570 Mühlheim, Germany; stefanie.grom@klsmartin.com (S.G.); tatjana.fecht@klsmartin.com (T.F.); janmarc.schehl@klsmartin.com (J.M.S.); tobias.wolfram@klsmartin.com (T.W.); frank.reinauer@klsmartin.com (F.R.); 4 Pulmonary Engineering Group, Department of Anesthesiology and Intensive Care Medicine, University Hospital Carl Gustav Carus at Technische Universität Dresden, 01307 Dresden, Germany; martin.scharffenberg@uniklinikum-dresden.de (M.S.); jakob.wittenstein@uniklinikum-dresden.de (J.W.); 5 INNOTERE GmbH, 01445 Radebeul, Germany; a.hoess@innotere.de (A.H.); s.heinemann@innotere.de (S.H.)

**Keywords:** additive manufacturing, Arburg Plastic Freeforming, 3D printing, poly(DL-lactide), calcium carbonate, strontium carbonate, tricalcium phosphate, hydroxyapatite, osteoblasts, bone defect

## Abstract

A promising therapeutic option for the treatment of critical-size mandibular defects is the implantation of biodegradable, porous structures that are produced patient-specifically by using additive manufacturing techniques. In this work, degradable poly(DL-lactide) polymer (PDLLA) was blended with different mineral phases with the aim of buffering its acidic degradation products, which can cause inflammation and stimulate bone regeneration. Microparticles of CaCO_3_, SrCO_3_, tricalcium phosphates (α-TCP, β-TCP), or strontium-modified hydroxyapatite (SrHAp) were mixed with the polymer powder following processing the blends into scaffolds with the Arburg Plastic Freeforming 3D-printing method. An in vitro degradation study over 24 weeks revealed a buffer effect for all mineral phases, with the buffering capacity of CaCO_3_ and SrCO_3_ being the highest. Analysis of conductivity, swelling, microstructure, viscosity, and glass transition temperature evidenced that the mineral phases influence the degradation behavior of the scaffolds. Cytocompatibility of all polymer blends was proven in cell experiments with SaOS-2 cells. Patient-specific implants consisting of PDLLA + CaCO_3_, which were tested in a pilot in vivo study in a segmental mandibular defect in minipigs, exhibited strong swelling. Based on these results, an in vitro swelling prediction model was developed that simulates the conditions of anisotropic swelling after implantation.

## 1. Introduction

The regeneration of bone tissue in critical-size defects caused, e.g., by tumor resection, is still an unsolved clinical problem. In the oral and maxillofacial region, the functional success of reconstructive surgical procedures is of particular importance to the patient, as numerous body functions (e.g., food and fluid intake, seeing, smell and taste, speech) are directly dependent on the correct anatomical structures. In addition, maintaining or restoring an aesthetically pleasing appearance is essential for interpersonal communication. Therefore, the possibility of producing patient-specific implants using additive manufacturing methods is of particular importance for oral and maxillofacial surgery. Though the process chain for CAD/CAM (computer-aided design and manufacturing) fabrication of individual implants based on clinical CT (computer tomography) or MRI (magnetic resonance imaging) data is established [1], clinical application is limited regarding suitable biomaterials. Non-biodegradable materials such as titanium and its alloys have significant disadvantages, as they impede complete tissue regeneration and permanently remain as foreign bodies in the organism that bear the risk of soft tissue damage. Therefore, research is focused on the development of biodegradable materials that are suitable for additive manufacturing of patient-specific implants.

Biomaterials based on polyester poly(lactic acid) (PLA) possess high biocompatibility and favorable mechanical properties and are already in clinical application for bone regeneration [2]. Their thermoplastic nature enables their processing into predefined structures by 3D printing techniques such as fused filament fabrication [3]. PLA exists in three isoforms consisting of either L-lactide, D-lactide, or racemic DL-lactide [4]. Poly(L-lactide) (PLLA) and poly(D-lactide) (PDLA) exhibit longer degradation times because of their (semi)crystallinity; the mixture of D- and L-isomers leads to the formation of amorphous poly(DL-lactide) (PDLLA), which is characterized by less tightly packed polymer chains and therefore faster degradation [2,5]. The first phase of degradation is based on hydrolysis of the long polymer chains into shorter fragments, which are phagocytosed and metabolized intracellularly in a second phase [5]. However, the shorter fragments, including lactic acid, decrease the pH value in the surrounding microenvironment. Especially in case of implants with larger dimensions, this acidification can induce an inflammatory reaction and damage cells necessary for tissue regeneration [6]. One promising strategy to reduce this negative effect is the blending of PLA-based biomaterials with inorganic nano/microparticles that have a buffer effect [7,8,9].

In this study, the clinically approved (FDA (U.S. Food and Drug Administration) K080862) polymer PDLLA was blended with different mineral phases with the aim of buffering its acidic degradation products and improving its bioactivity. A first systematic comparison of mineral phases with different solubilities and acidity/basicity (CaCO_3_, SrCO_3_, tricalcium phosphates (α-TCP, β-TCP), or strontium-modified hydroxyapatite (SrHAp)) as fillers was carried out in our previous study for the copolymer PLLA-PGA (poly(L-lactic-co-glycolic) [7]; in this work, we investigated the effect of the mineral fillers for the amorphous polymer PDLLA, which has great potential in bone regenerative procedures [5]. The blends were 3D-printed using the additive manufacturing method Arburg Plastic Freeforming [10], and the resulting scaffolds were thoroughly characterized regarding their degradation behavior and their cytocompatibility in comparison to pure PDLLA scaffolds. Finally, a first proof of concept was performed in a pilot in vivo experiment examining the behavior of a patient-specific implant printed from PDLLA blended with CaCO_3_ microparticles in a segmental mandibular defect in minipigs.

## 2. Materials and Methods

### 2.1. Materials and Scaffold Fabrication

PDLLA was obtained as pellets from Evonik Industries (Essen, Germany). Microparticles of mineral phases were the same as described recently [7]: Particles of SrCO_3_, SrHAp (obtained from a set strontium-containing calcium phosphate cement [11]), α-TCP, and β-TCP were provided by INNOTERE, (Radebeul, Germany). Particles of CaCO_3_ were obtained from Schaefer Kalk (Diez, Germany).

**Figure 1:**
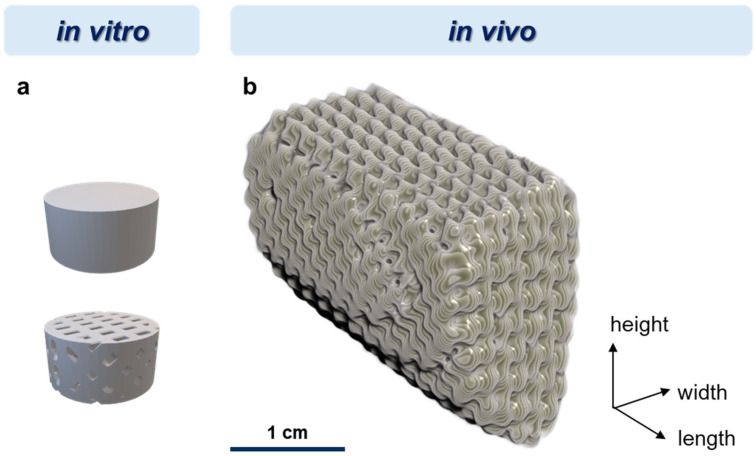
Design of 3D-printed porous and bulk scaffolds used for the in vitro analyses of degradation and cytocompatibility, respectively (**a**), and one of the two implants for the in vivo analysis (**b**) with approximate dimensions of 29 × 20 × 20 mm (length × width × height). Exact dimensions were dependent on the individual DVT data.

**Figure A1:**
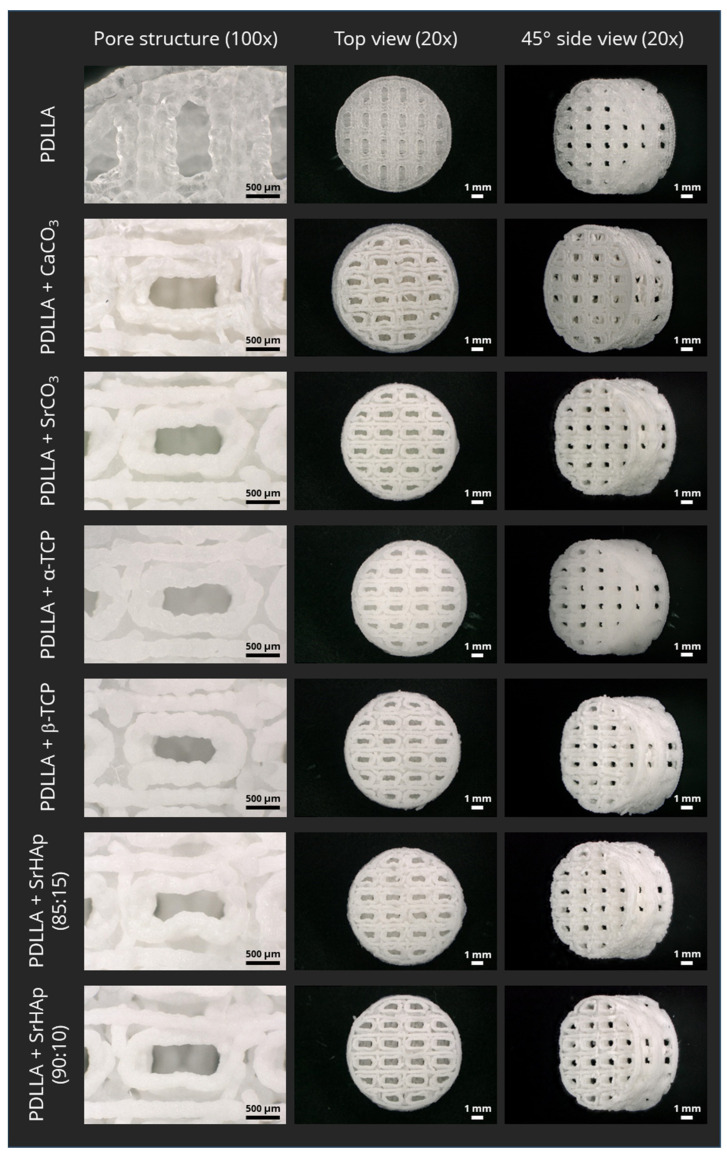
Scaffold fabrication: porous samples for in vitro analysis. Three-dimensional printed porous scaffolds of PDLLA and PDLLA blended with various mineral phases used for the in vitro degradation experiment. Light microscopic images taken at week 0 of the degradation experiment (scaffold dimensions: diameter of 10 mm, height of 5 mm, designed with a strand thickness of 1 mm and pore dimensions in x and y directions of 1.3 mm and 0.6 mm, respectively (diagonal ca. 1.4 mm)).

The PDLLA powder and the mineral particles were mixed in a mass ratio of 85:15 (PDLLA:particles); an additional mass ratio of 90:10 was used for the SrHAp particles. The mixtures were ground using a speed mixer (Hauschild, Hamm, Germany) at 500–1000 rpm at 20 °C, then pressed into a block at temperatures of 150–200 °C, granulated in a cutting mill (Retsch, Haan, Germany) at 2000–3000 rpm, and dried under vacuum (<50 mbar) for >6 h at 20 °C. For scaffold fabrication, the 3D printing system Freeformer (Arburg, Lossburg, Germany) was used. For the in vitro analyses, cylindrical bulk and porous samples with a diameter of 10 mm and a height of 5 mm were fabricated at an ambient temperature of 40–60 °C and a melt pressure of 30 MPa; the porous samples were designed with a strand thickness of 1 mm and pore dimensions in x and y directions of 1.3 mm and 0.6 mm, respectively (diagonal ca. 1.4 mm) (Figure 1a; Figure A1 in Appendix A). For the in vivo testing, individual implants (scaffolds, osteosynthesis plates) and cutting guides were fabricated based on X-ray (digital volume tomography; DVT) data (see Section 2.4). Therefore, the lower jaw of each individual animal was scanned using DVT. DVT data were imported into Geomagic Freeform software (Artec 3D, Senningerberg, Luxemburg) and processed, and implants were modeled and designed individually, with an inner geometry similar to the porous in vitro samples (Figure 1b). Whereas PDLLA-based scaffolds and cutting guides were printed using a Freeformer 200-3X (Arburg, Lossburg, Germany), osteosynthesis plates were produced using an SLM 125 printer (NIKON SLM Solutions, Lübeck, Germany). After fabrication, scaffolds and cutting guides were vacuum dried for storage, and all samples were then sterilized by gamma irradiation (max. 25 kGy).

### 2.2. Aging Experiment and Analysis of Degradation

Porous samples were incubated in deionized water at 37 °C (4 scaffolds at 0.25 g = 1 g in 50 mL; in total, 20 scaffolds were analyzed) over 24 weeks, referring to DIN EN ISO 10993-13 [12].

#### 2.2.1. Measurement of Dimensional Change

Dimensional changes were determined at weeks 0, 2, 4, and 24 by measurement of the outer scaffold dimensions (height, diameter) and the thickness of the strands in the x (horizontal) and y (vertical) directions using a Keyence (Osaka, Japan) VHX-6000 digital microscope (n = 3).

#### 2.2.2. Scanning Electron Microscopy

The morphology of the strand surfaces at different time points of degradation was analyzed by scanning electron microscopy (SEM). Scaffolds were dried, gold sputtered using a Sputter Coater (JFC-1200 Fine Coater; Jeol, Peabody, MA, USA), and imaged by SEM (EVO 25; Zeiss, Oberkochen, Germany).

#### 2.2.3. Measurement of pH Value and Conductivity

Development of the pH and the conductivity in the incubation batches (n = 3) was monitored by measuring the pH values after sterilization (week 0) and then every second week until week 24 with a pH/conductivity meter (Multi 9260 IDS with pH electrode Sentix 980 IDS and conductivity electrode TetraCon 925; WTW, Xylem Analytics, Weilheim, Germany).

#### 2.2.4. Differential Scanning Calorimetry

At different time points of degradation (at weeks 0, 2, 4, and 24), desiccator-dried samples (n = 2) were analyzed by differential scanning calorimetry (DSC; 214 Polyma; Netzsch, Selb, Germany) in accordance with DIN EN ISO 11357-3 [13]. The samples were heated from −20 °C to 210 °C at a heating rate of 10 K/min (first heating), cooled down at a cooling rate of 10 K/min, and heated again from −20 °C to 210 °C at a heating rate of 10 K/min (second heating). The flow rate of nitrogen gas was 60 mL/min.

#### 2.2.5. Measurement of Viscosity

Viscosity was measured in accordance with DIN EN ISO 1628 [14]. At different time points of degradation (at weeks 0, 2, 4, and 24), scaffolds (n = 2) were dried using a desiccator and dissolved in 100% (*w*/*v*) chloroform with a ratio of 50 mL solvent per 50 mg material. Inherent viscosity was measured using an Ubbelohde viscosimeter (PVS, Lauda Scientific; Königshofen, Germany) (K = 0.005 mm^2^/s^2^); measurement was repeated two times.

### 2.3. Cell Culture Experiments

The osteoblast-like cell line SaOS-2 (ATCC 243; DSMZ, Braunschweig, Germany) was used to study the cytocompatibility of the polymer blends in direct contact culture as described previously [7]. In brief, the cells were expanded in cell culture medium consisting of Minimum Essential Medium alpha modification (αMEM; Gibco, ThermoFisher Scientific, Waltham, MA, USA), 15% fetal calf serum (FCS; Corning, NY, USA), and 100 U mL^−1^ penicillin and 100 μg mL^−1^ streptomycin (PS). Bulk samples, pre-incubated in cell culture medium overnight, were seeded with 4 × 10^4^ cells. After initial cell attachment for 24 h, the seeded scaffolds were transferred to fresh cell culture plates and incubated in cell culture medium with or without the osteogenic supplements (10^−7^ M dexamethasone, 50 µM ascorbic acide-2-phosphate, 10 mM β-glycerophosphate; all from Sigma-Aldrich, Germany); change of medium was performed twice a week. After 1, 7, and 14 days of culture, samples were taken, washed with phosphate-buffered saline (PBS; ThermoFisher Scientific, USA), and either stored at −80 °C until biochemical analysis or fixed in 4% formaldehyde in PBS for microscopical analysis.

For quantification of cell number and alkaline phosphatase (ALP) activity, the thawed samples were incubated in 500 µL lysis buffer (1% Triton X-100 in PBS) for 50 min on ice. DNA was quantified in the lysates using the FluoReporter™ Blue Fluorometric dsDNA Quantitation Kit (Invitrogen^TM^, ThermoFisher Scientific, USA) according to the manufacturer’s instructions; fluorescence was measured at Ex/Em 360/460 nm with a microplate reader (Infinite^®^ M200 Pro, Tecan, Zürich, Switzerland). Correlation of the DNA amount with the total cell number was achieved using a calibration line obtained from defined cell numbers. Lactate dehydrogenase (LDH) activity was determined in the lysates using the CytoTox 96^®^ Non-Radioactive Cytotoxicity Assay (Promega, Madison, WI, USA) according to the manufacturer’s instructions by measuring the absorbance at 490 nm (Infinite^®^ M200 Pro) and correlated with the number of metabolically active cells using a calibration line from the LDH activity of defined cell numbers. The activity of alkaline phosphatase (ALP) was determined as described recently [7]. One aliquot of the cell lysates was incubated with ALP substrate solution for 10 min at 37 °C; the absorbance was read at 405 nm (Infinite^®^ M200 Pro) after stopping the enzymatic reaction with 1 M NaOH. Another aliquot of the same lysate was used for DNA quantification, and specific ALP activity was calculated by correlating the absorbance to a p-nitrophenol calibration line and the respective DNA-derived cell number. Fluorescence microscopical analysis of cell adhesion, morphology, and density was carried out as described recently [7]. In brief, after staining with DAPI (Sigma-Aldrich, Taufkirchen, Germany) and Alexa Fluor 488 phalloidin (Invitrogen^TM^, ThermoFisher Scientific, USA) for visualization of cell nuclei and actin cytoskeletons, the samples were imaged using a Keyence BIOREVO BZ-X800 fluorescent microscope (Keyence, Japan).

### 2.4. Pilot In Vivo Study

#### 2.4.1. Study Design

**Figure A2:**
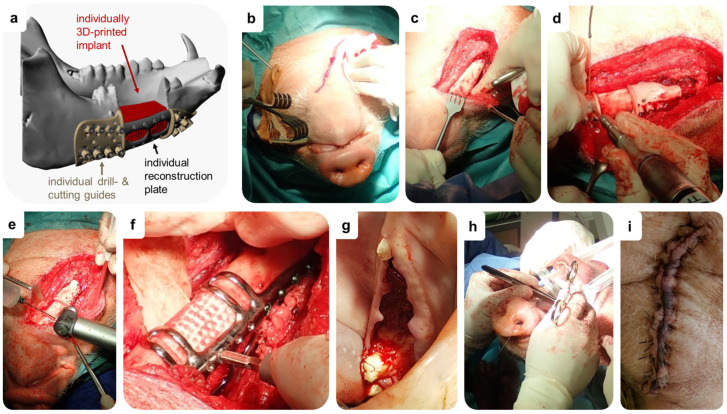
Pilot in vivo study. Segmental mandibular defect model used to test individually manufactured scaffolds (**a**). Surgical procedure: skin incision (**b**), dissection of soft tissue and periosteum to expose the lower jawbone (**c**), application of individually produced cutting and drill guides followed by drilling of the holes for fixing the osteosynthesis plate (**d**), osteotomy (**e**), application of individually manufactured osteosynthesis plate and PDLLA + CaCO_3_ scaffold followed by fixing of the scaffold to the reconstruction plate (**f**), closing of intra- and extraoral incisions in multilayer fashion (**g**–**i**).

For in vivo testing, a segmental lower jawbone defect was created in 2 adult minipigs, filled with individually manufactured implants made of PDLLA + CaCO_3_ and stabilized by an individual titanium reconstruction plate (Figure A2a in Appendix A). During the 6-month-long observation period, the progress of bone regeneration was monitored by repetitive DVT scans. After 6 months, animals were sacrificed, and explanted specimens were analyzed macroscopically and by histology.

#### 2.4.2. Animals and Ethics Statement

Two skeletally mature, castrated male Aachener minipigs (minipig #1: 1.8 years, 65 kg; minipig #2: 1.7 years, 51 kg) were purchased from Heinrichs Genetics GmbH (Waldfeucht, Germany) and housed in stables for 10 days for acclimatization prior to any experiments. While being kept at a 12 h light–dark cycle, animals were fed twice a day with pelleted commercial food and maintained ad libitum with water. After the operations, food was mixed with water to facilitate food intake and to minimize mechanical load. All surgical procedures, housing, and animal care were carried out in accordance with the German legislation for animal protection and the regulations for animal experiments of the state of Saxony. The trials were approved by the local animal committee of the District Government of Saxony, Dresden, Germany (Approval No: 25-5131/496/46).

#### 2.4.3. DVT Measurements

DVT scans (120 kV, 57.6 mAs, voxel size = 400 µm; xCAT, Xoran, Ann Arbor, MI, USA) of the skull of each animal were performed under general anesthesia via intramuscular injection of midazolam (1 mg/kg; Ratiopharm, Ulm, Germany), ketamine (10 mg/kg; Inresa Arzneimittel GmbH, Freiburg, Germany), and atropine (0.05 mg/kg; B. Braun, Melsungen, Germany). Pre-operative scans were performed to provide anatomical data for the production of individual implants (scaffolds, osteosynthesis plates, cutting guides), and postoperative scans at 0, 2, 4, and 6 months were performed to assess bone regeneration. DVT data were analyzed using Dragonfly ORS (version 2021.1.0.977, Comet Technologies Canada Inc., Montreal, QC, Canada).

#### 2.4.4. Surgical Procedure for Implantation of Individual Implants

The surgery was performed under general anesthesia. For stressless transport to the operation room, the minipigs received midazolam (1 mg/kg; Ratiopharm, Ulm, Germany), ketamine (10 mg/kg; Inresa Arzneimittel GmbH, Freiburg, Germany), and atropine (0.05 mg/kg; B. Braun, Melsungen, Germany) via intramuscular injection. To enable endotracheal intubation, animals were anesthetized using intravenous ketamine (15 mg/kg/h) and midazolam (1 mg/kg/h), orotracheally intubated, placed in a supine position, and ventilated according to clinical recommendations for intra-operative lung-protective ventilation. Basic cardio-respiratory monitoring was established, and vital signs were continuously controlled throughout surgery. Fluid balance was maintained using intravenous balanced crystalloids (10 mL/kg/h). Additionally, intraoperative analgesics (Carprofen; 4 mg/kg, Rimadyl, Zoetis Deutschland GmbH, Berlin, Germany) and prophylactic antibiotics were administered to reduce the risk of infection (Amoxicillin; 15 mg/kg, Duphamox L.A., Zoetis Deutschland GmbH).

Following anesthetic induction, the surgical site was disinfected and isolated with sterile drapes. A submandibular S-shaped skin incision was made a few centimeters next to the contour of the right lower jawbone (Figure A2b in Appendix A), followed by soft tissue and periosteum dissection to expose the mandible (Figure A2c in Appendix A). The individually manufactured cutting and drill guides were fixed to the bone, and holes for fixing the osteosynthesis plate were drilled (Figure A2d in Appendix A). Using an oscillating bone saw cooled with sterile saline, osteotomy was performed along the borders of the cutting guides to create the segmental defect (Figure A2e in Appendix A). To ensure stability and prevent mandibular fractures, an individually produced titanium reconstruction plate (KLS Martin SE & Co. KG, Mühlheim, Germany) was applied at the lateral side of the mandible. After inserting the individually 3D-printed PDLLA + CaCO_3_ scaffold (KLS Martin SE & Co. KG; length × width × height animal #1: 29 × 19 × 23 mm, animal #2: 29 × 20 × 20 mm), it was fixed to the osteosynthesis plate with SonicPins Rx^®^ (KLS Martin SE & Co. KG; Figure A2f in Appendix A). Finally, the incisions were closed intra- and extraorally in a multilayer fashion (Vicryl Plus 0, Ethicon, Norderstedt, Germany; Figure A2g–i in Appendix A), and the skin wound was disinfected.

After completing surgery and performing the first postoperative DVT scan (0 month), sufficient spontaneous breathing was resumed, and pigs were extubated under stable cardio-respiratory conditions. For postoperative pain management, the minipigs received fentanyl transdermally (plaster, 12 µg/h; Ratiopharm, Ulm, Germany) for 3 days and Carprofen orally (4 mg/kg/d) for 10 days. Infection prophylaxis was provided orally using Amoxicillin (1 tablet Amoclav, 875 mg Amoxicillin + 175 mg clavulanic acid per day; Hexal, Holzkirchen, Germany) for 10 days. Six months after implantation, the minipigs were sacrificed under general anesthesia by intravenous bolus injection of 2 g thiopental (Inresa Arzneimittel GmbH, Freiburg, Germany) and 50 mL 1 M potassium chloride (Fresenius Kabi, Bad Homburg, Germany) in accordance with the ethical standards. The mandible was harvested, examined, and prepared for histologic evaluation.

### 2.5. In Vitro Swelling Model for Prediction

The 3D model for simulation of anisotropic swelling was fabricated by fused deposition modeling using an MK3 printer and “Prusament” PLA filament as printing material (Prusa, Prague, Czech Republic). The cavity in which the porous scaffolds were “loose-fit” had a diameter of 10.2 mm and a height of 5 mm. The channel allowing directional swelling had a width of 3.7 mm. The outer diameter of the 3D model was 33.7 mm. For swelling analysis, the 3D models were disinfected in 70% ethanol and placed in 6-well cell culture plates. The scaffolds were positioned in the middle of the cavity, and 1 mL incubation medium was added, completely covering the 3D model with the inserted scaffold. As incubation medium, 0.9% NaCl, 0.9% NaCl + HCl (pH 4.2), and cell culture medium (αMEM with 9% FCS and PS) were used. The incubation was carried out at 37 °C for max. 9 weeks; the incubation medium was refreshed once per week. First at day 0 and then every week (7-day cycle), the 3D models with inserted scaffolds were imaged using a stereo light microscope (M205C equipped with a DFC295 camera; Leica, Berlin, Germany). The images were analyzed by determining the anisotropic quotient, which indicates the ratio of the scaffold widths in the open direction (of the channel) and in the closed direction (perpendicular to the channel) as well as by measuring the diagonal pore diameters using Fiji software (version ImageJ 1.53n) [15].

### 2.6. Statistics

The data of quantitative measurements are depicted as mean ± standard deviation. All values were evaluated either by one-way or two-way analysis of variance (ANOVA) depending on the data, followed by multiple comparison tests with GraphPad Prism 10 software. Values of *p* < 0.05 were considered significant.

## 3. Results

### 3.1. Influence of the Mineral-Phase Microparticles on Degradation

#### 3.1.1. Dimensional Change

**Figure 2:**
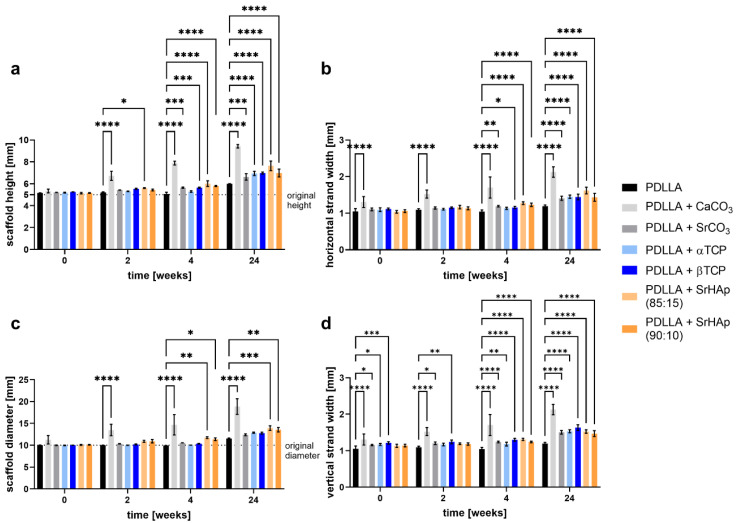
Dimensional change in 3D-printed porous scaffolds consisting of PDLLA blended with different mineral phases in comparison to pure PDLLA during aging in water over 24 weeks: average scaffold height (**a**) and diameter (**c**); average strand width in x- (**b**) and y-direction (**d**); (n = 3, mean ± SD, significant differences of PDLLA vs. the blends: * *p* < 0.05, ** *p* < 0.01, *** *p* < 0.001, **** *p* < 0.0001). The dotted line in (**a**,**c**) represents the dimension according to the scaffold design.

During aging of porous scaffolds in water over 24 weeks, scaffold dimensions increased depending on the material composition (Figure 2). For pure PDLLA scaffolds, no change in scaffold height, diameter, or strand width was observed within the first 4 weeks, and only a marginal increase was seen until week 24. A moderate increase, which already occurred in the first 4 weeks and persisted until week 24, was measured for scaffolds of PDLLA blended with SrCO_3_, α-TCP, β-TCP, and SrHAp. The highest increase in scaffold height and diameter as well as in strand width was exhibited in the scaffolds consisting of PDLLA + CaCO_3_.

#### 3.1.2. Morphology of Strand Surface

**Figure 3:**
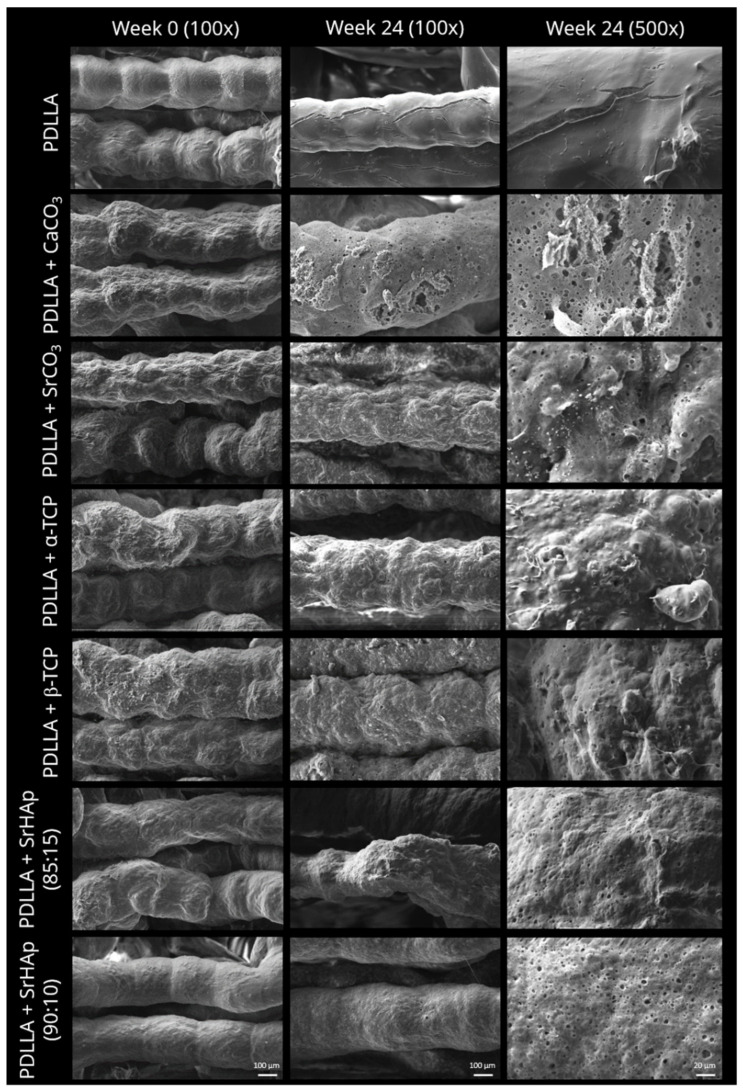
Scanning electron microscopic images of 3D-printed porous scaffolds consisting of pure PDLLA or PDLLA blended with different mineral phases before (week 0) and after aging in water over 24 weeks; scale bars represent 100 µm (100x) and 20 µm (500x).

Scanning electron microscopic analysis of porous scaffolds revealed that after fabrication and sterilization (week 0), the strand surface of PDLLA blended with mineral microparticles appeared slightly rougher compared to those of pure PDLLA samples (Figure 3). The PDLLA + SrHAp scaffolds indicated a correlation between the amount of particles and the surface appearance, as samples with 10 wt% particles exhibited a smoother surface than samples with 15 wt% particles. After 24 weeks of aging, pores were observed on the strand surface in all particle-containing groups; the number and size of these micropores appeared to be highest for PDLLA + CaCO_3_ scaffolds (Figure 3). In contrast, pure PDLLA scaffolds exhibited a smooth and dense surface with a few cracks.

#### 3.1.3. Development of pH and Conductivity

**Figure 4:**
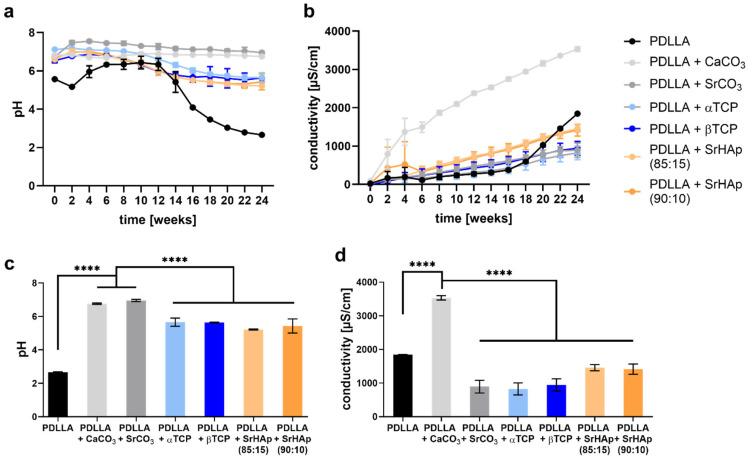
Measurement of pH value (**a**) and conductivity (**b**) in the supernatant of 3D-printed porous scaffolds consisting of pure PDLLA or PDLLA blended with different mineral phases during aging in water over 24 weeks; highly significant differences in pH value (**c**) and conductivity (**d**) at week 24 (n = 3, mean ± SD, **** *p* < 0.0001).

Monitoring of the pH value in the supernatant over 24 weeks revealed that all mineral phases used for blending are able to buffer the acidic degradation products of PDLLA: Only for the pure PDLLA scaffolds a sharp drop in pH was observed after week 12, which ended below pH = 3 at week 24 (Figure 4a). The highest buffering capacity exhibited the carbonate phases: the average pH value measured for the blends PDLLA + CaCO_3_ and PDLLA + SrCO_3_ was around pH = 7 over the whole observation period. The blends containing phosphate phases showed a slight decrease of the average pH value from around pH = 7 at week 0 to pH = 6 at week 24. At week 24, pH values were significant higher in the groups of PDLLA blended with CaCO_3_ or SrCO_3_ compared to pure PDLLA as well as PDLLA blended with phosphate phases (Figure 4c).

The conductivity was measured in the supernatant as indicator of degradation based on ion release (Figure 4b). The strongest increase over the 24 weeks was observed for the PDLLA + CaCO_3_ blend, indicating the highest degradation rate of the mineral phase. Conductivity of PDLLA blended with SrCO_3_, α-TCP and β-TCP increased slowly over time; PDLLA blended with SrHAp exhibited a somewhat higher conductivity. The pure PDLLA samples showed a slow increase until week 16 followed by a strong increase until the end of the observation period. At week 24, conductivity was significantly higher in the group of PDLLA + CaCO_3_ compared to pure PDLLA as well as PDLLA blended with SrCO_3_ or the phosphate phases (Figure 4d).

#### 3.1.4. Differential Scanning Calorimetry

**Figure 5:**
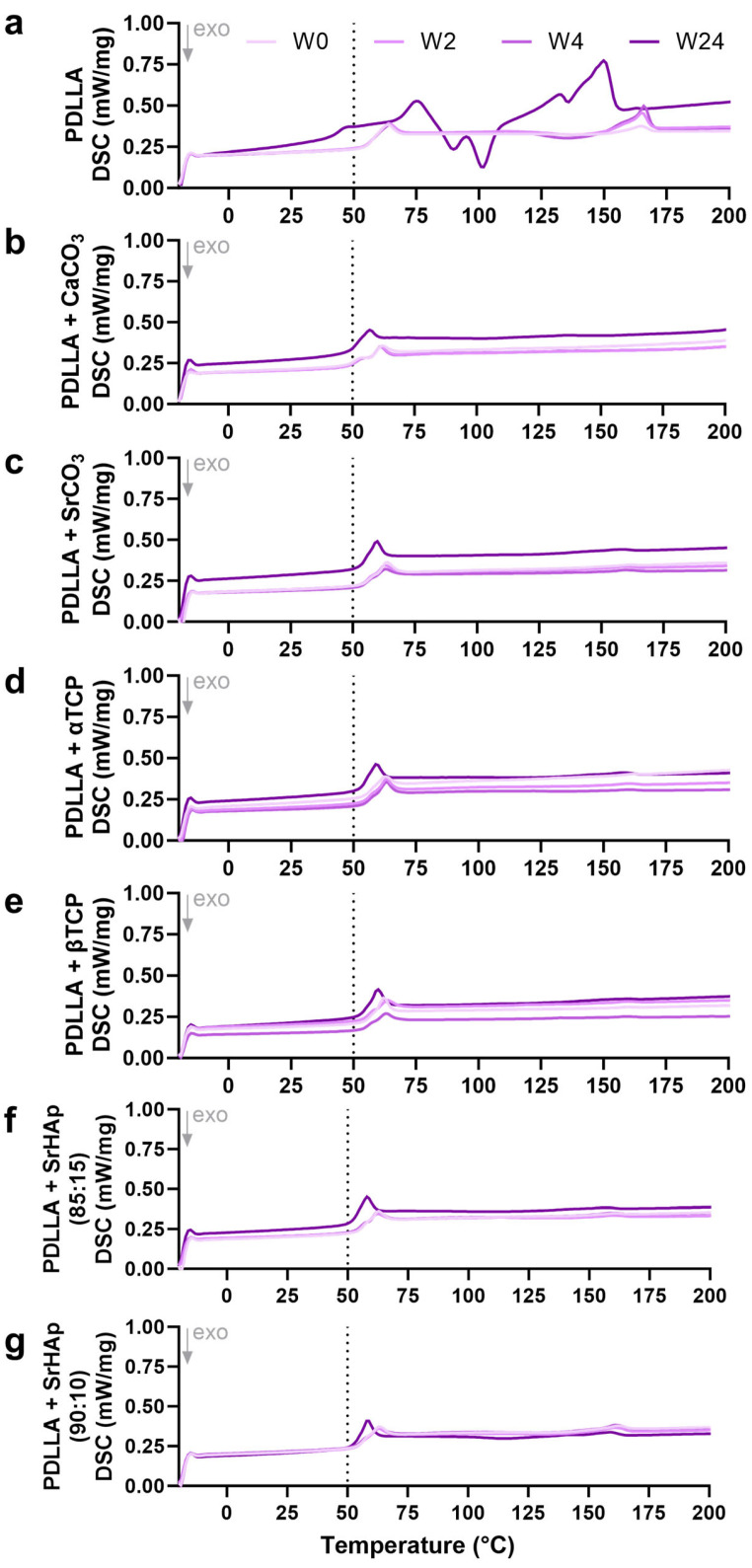
Differential scanning calorimetry of 3D-printed porous scaffolds consisting of pure PDLLA (**a**) or PDLLA blended with different mineral phases (**b**–**g**) before (week 0 (W0)) and at various time points of aging (weeks 2 (W2), 4 (W4), 24 (W24)) in water. The glass transition temperature is in the range of 50–60 °C.

A first heating phase was conducted to allow for rearrangement of the amorphous regions and thus to eliminate the thermal history of the polymer [16]. In the second heating phase, the glass transition temperature (T_g_), whose decrease indicates polymer degradation, can be identified. For the pure PDLLA samples, a strong shift in T_g_ from ≈65 °C in the early phase of incubation (weeks 0, 2, 4) to < 50 °C at week 24 of aging was observed (Figure 5a). In contrast, for the blended PDLLA groups, a much lower decrease of T_g_ to ≈50–60 °C was measured, indicating a reduced degradation in the presence of the mineral phases (Figure 5b–g). At higher temperatures (around 100 °C and higher), pure PDLLA seemed to recrystallize during degradation, indicated by crystallization (negative) peaks and melt (positive) peaks (Figure 5a); recrystallization was not observed for the blend groups (Figure 5b–g).

#### 3.1.5. Changes in Viscosity

**Figure 6:**
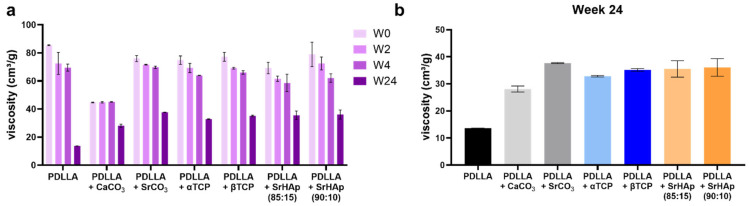
Measurement of the inherent viscosity of 3D-printed porous scaffolds consisting of pure PDLLA or PDLLA blended with different mineral phases before (week 0 (W0)) and at various time points of aging (weeks 2 (W2), 4 (W4), 24 (W24)) in water (**a**); differences in viscosity at week 24 (**b**) (n = 2, mean and minimum as well as maximum value are depicted).

Viscosity measurement of the samples revealed a decrease over the 24 weeks of incubation for all groups, indicating degradation of the polymer (Figure 6a). The pure PDLLA group exhibited the highest viscosity initially (week 0), but during aging, it exhibited the strongest decrease in viscosity; after 24 weeks, viscosity of all blended polymer groups was significantly higher compared to pure PDLLA (Figure 6b). The PDLLA + CaCO_3_ group again showed a divergent behavior, since its viscosity was initially lower compared to all other groups and remained constant at this level during the early phase of degradation (Figure 6a).

### 3.2. Analysis of Cytocompatibility

**Figure 7:**
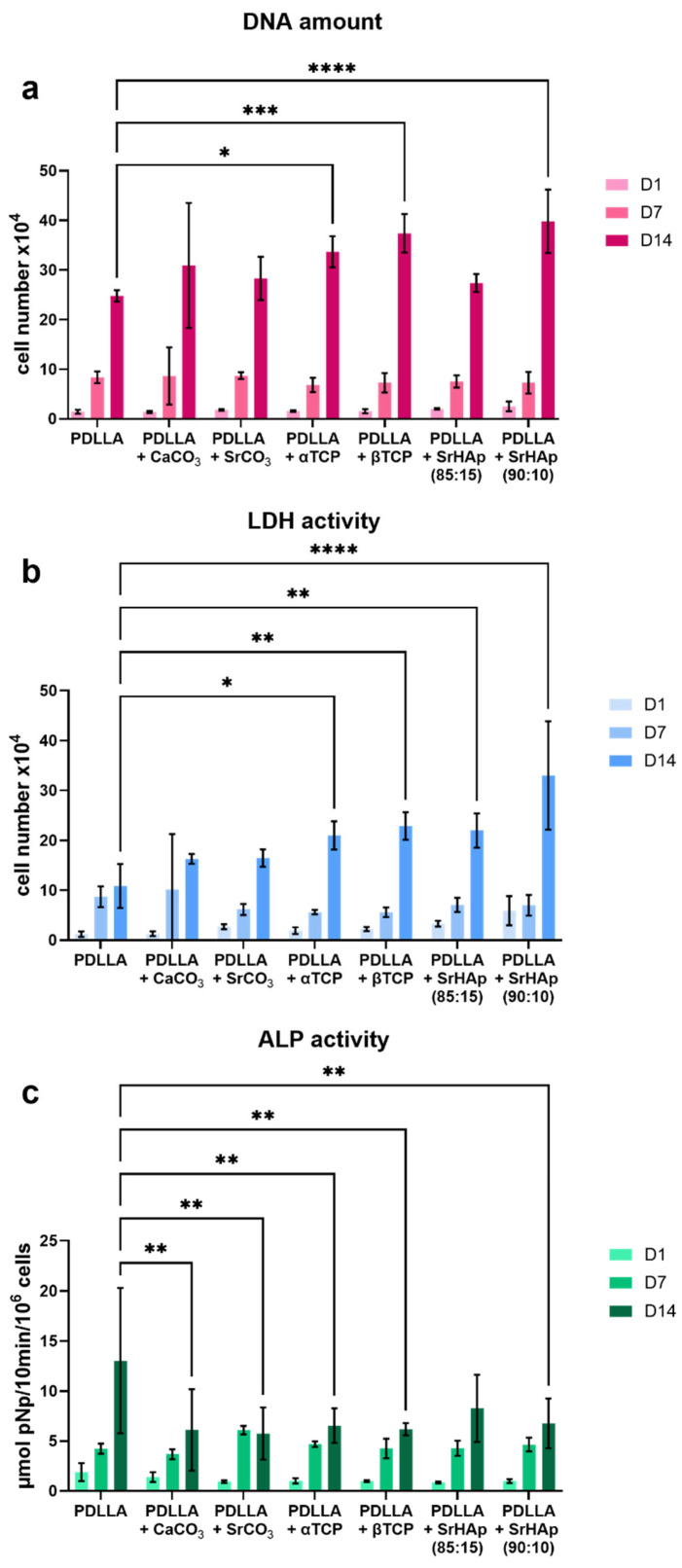
Cultivation of SAOS-2 cells on 3D-printed bulk scaffolds consisting of PDLLA blended with different mineral phases in comparison to pure PDLLA: total cell number was correlated with the DNA amount measured after cell lysis (**a**); the number of metabolically active cells was correlated with the cytosolic LDH activity measured after cell lysis (**b**); the ALP activity as indicator of osteogenic differentiation is shown as specific activity in relation to the cell number (**c**); for analysis of ALP activity, the cell-seeded samples were cultured in medium with osteogenic supplements; (n = 3, mean ± SD, significant differences of PDLLA vs. the blends: * *p* < 0.05, ** *p* < 0.01, *** *p* < 0.001, **** *p* < 0.0001).

**Figure A3:**
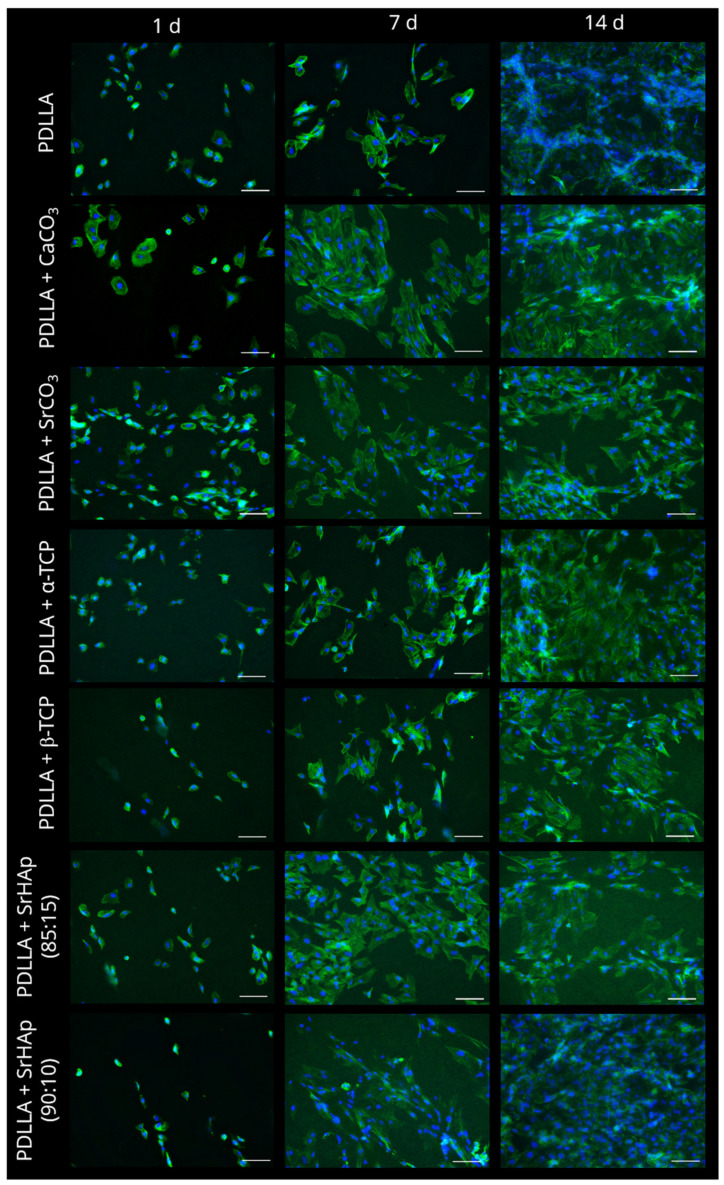
Analysis of cytocompatibility. Fluorescence microscopic images of SAOS-2 cells on 3D-printed bulk scaffolds consisting of pure PDLLA and PDLLA blended with different mineral phases. After seeding, the samples were incubated over 14 days in cell culture medium without osteogenic supplements. Cytoskeletons are stained with Alexa Fluor 488 Phalloidin (green), and cell nuclei are stained with DAPI (blue). Scale bars represent 100 µm.

Cell culture experiments with SaOS-2 cells, seeded and cultivated on the printed scaffolds, were performed to assess the cytocompatibility of PDLLA blended with different mineral phases in comparison to pure PDLLA (Figure 7). An increase in the total cell number over the cultivation period of 14 days, calculated from the DNA content, was observed for all groups; on day 14, a significantly higher cell number was determined in the groups of PDLLA blended with α-TCP, β-TCP, and SrHAp (90:10) compared to pure PDLLA (Figure 7a). Measurement of cytosolic LDH activity, reflecting the number of metabolically active cells, also showed an increase in all groups over the cultivation period. In comparison to pure PDLLA, significantly higher numbers of metabolically active cells were observed for PDLLA blended with the phosphate phases on day 14 (Figure 7b). In all groups, the cells cultured on scaffolds showed an increase in the specific ALP activity, a characteristic marker of the osteogenic phenotype, over the cultivation time. Except for PDLLA + SrHAp (85:15), the ALP activity on day 14 was significantly lower on the PDLLA-based blends than on pure PDLLA (Figure 7c). Fluorescence microscopic analysis of the cell-seeded scaffolds confirmed that the cells attached and proliferated on all scaffold variants (Figure A3 in Appendix A). On day 1, the cells exhibited an initial spread morphology, which appeared to be most pronounced on PDLLA + CaCO_3_. On day 7, distinct cell spreading was observed on all scaffolds, and on day 14, the cells covered the surface nearly completely in all groups, with the highest coverage rate observed on PDLLA + CaCO_3_, PDLLA + SrHAP (90:10), and PDLLA.

### 3.3. Pilot In Vivo Experiment

Among all material variants tested in vitro, PDLLA blended with CaCO_3_ as well as with SrCO_3_ displayed the best buffer effect. Since the application of strontium in humans could cause regulatory problems due to its possible pharmacological effects on bone metabolism [17,18], and since the blend with CaCO_3_ appeared to degrade faster than the other blends, PDLLA + CaCO_3_ was chosen to produce the individual scaffolds for the in vivo pilot experiment. To account for swelling that was observed in the in vitro experiments, scaffolds to be used for implantation into the minipigs were not produced as a “perfect fit” into the defect, but with reduced size in the cranial direction to minimize pressure onto the sutured oral mucosa (Figure A2a, animal #1: height reduced by 10 mm, animal #2: height reduced by 17 mm).

**Figure 8:**
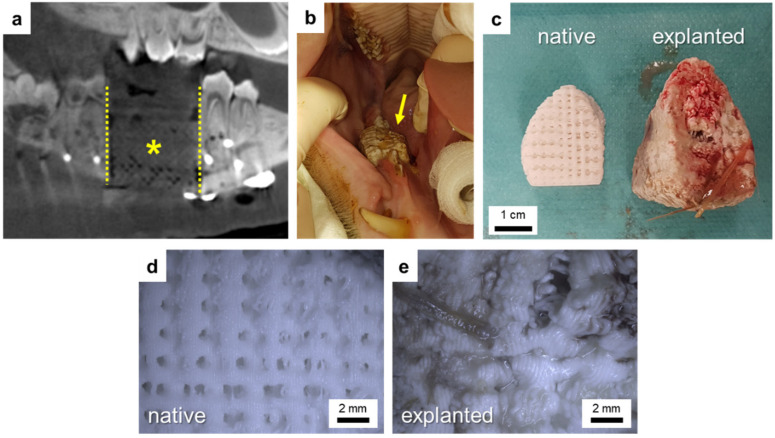
Minipig #1: good fit of the scaffold into the segmental mandibular defect as verified by X-ray analysis directly after implantation (**a**) (dotted lines = osteotomy borders, * = scaffold); examination at 6 weeks postoperation showed a large intraoral dehiscence (**b**) (arrow = dehiscence/swollen scaffold) due to a swollen scaffold (**c**–**e**).

**Figure 9:**
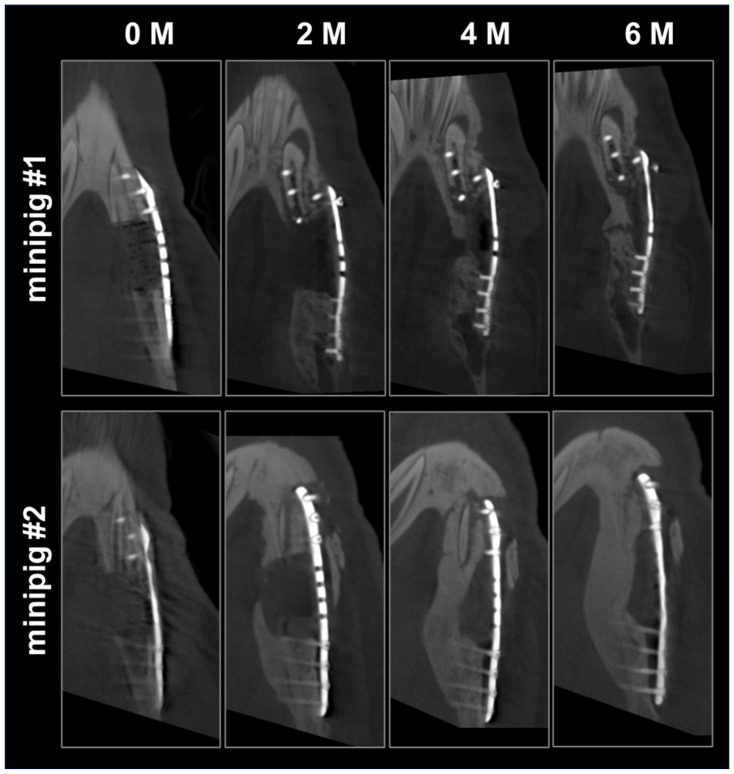
New bone formation after 0, 2, 4, and 6 months as analyzed by DVT measurement (top view of the defect area containing the reconstruction plate on the right side).

DVT scans performed directly after implantation showed good fit of the scaffold into the defect (Figure 8a). At 5 weeks postoperatively, both animals showed intraoral dehiscences that increased during the following week. Thus, 6 weeks postoperatively, the scaffold of animal #1 had to be removed due to strong swelling (Figure 8c–e). In order to preserve the scaffold of animal #2, it was manually ablated, and the oral mucosa was mobilized and sutured again, but at 8 weeks postoperatively, this scaffold also had to be removed completely due to swelling and large intraoral dehiscences. After scaffold removal, the intraoral wound was closed again, and both animals received analgesics and antibiotics for pain and infection management and were housed until the end of the 6-month observation period. As seen from DVT measurements, bone regeneration started from the osteotomy borders along the reconstruction plate and around the scaffold (Figure 9). After 6 months, the defect in minipig #1 was nearly bridged, and the one in minipig #2 was completely bridged by new bone. Thereby, a higher volume of new bone within the defect area could be detected for minipig #2 compared to minipig #1 (Figure 9).

### 3.4. Development of an In Vitro Degradation Model for Swelling Prediction

Based on the experiences of the pilot in vivo testing, an experimental model was established that is able to predict the swelling behavior of the printed, macroporous scaffolds in vivo. A 3D model was additively manufactured, into which a cylindrical sample was inserted (“implanted”) and in which the scaffold cannot swell in all directions, but instead emerge from the “defect” at two predefined sites (the channel entrances), simulating the anisotropic swelling situation after implantation in vivo. A possible influence of serum proteins and the surrounding pH value was considered by investigating the swelling comparatively in cell culture medium (buffered at pH 7.4 and with serum proteins), 0.9% NaCl (unbuffered, protein-free), and 0.9% NaCl + HCl (pH 4.2 to simulate acidification as a result of inflammation, protein-free).

**Figure 10:**
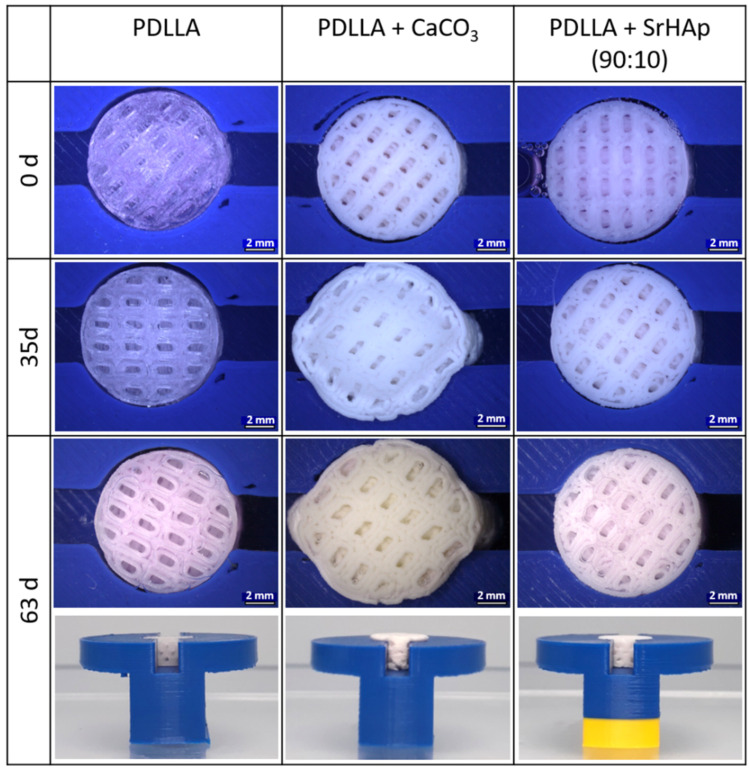
Anisotropic swelling of 3D-printed porous scaffolds consisting of PDLLA, PDLLA + CaCO_3_, and PDLLA + SrHAp (90:10) in a 3D-printed model for simulation of the situation after implantation in a bone defect (scale bar: 2 mm). Incubation was carried out in cell culture medium at 37 °C.

In a first attempt, swelling of PDLLA + CaCO_3_ was compared to pure PDLLA and PDLLA + SrHAp (90:10) as an example of one of the other blends. Figure 10 shows exemplarily images of samples taken at day 0 and after incubation in cell culture medium at 37 °C over 35 and 63 days (5 and 9 weeks, respectively). No swelling was observed for the pure PDLLA scaffold: the “loose fit”, visible by a small gap around the scaffold, was maintained even after 63 days of incubation in all three media. The PDLLA + CaCO_3_ scaffolds showed dramatic swelling during incubation, confirming the observations of the pilot in vivo test. The PDLLA + SrHAp (90:10) scaffolds exhibited marginal swelling: at day 35, the gap around the scaffolds was reduced, and it vanished at day 63. Quantification of the surface area of the top side of the cylindrical scaffolds is summarized in Table 1 for all investigated time points of incubation in cell culture medium; similar values were obtained for the two other incubation media (0.9% NaCl: Table A1 in Appendix A; 0.9% NaCl + HCl (pH 4.2): Table A2 in Appendix A).

**Figure 11:**
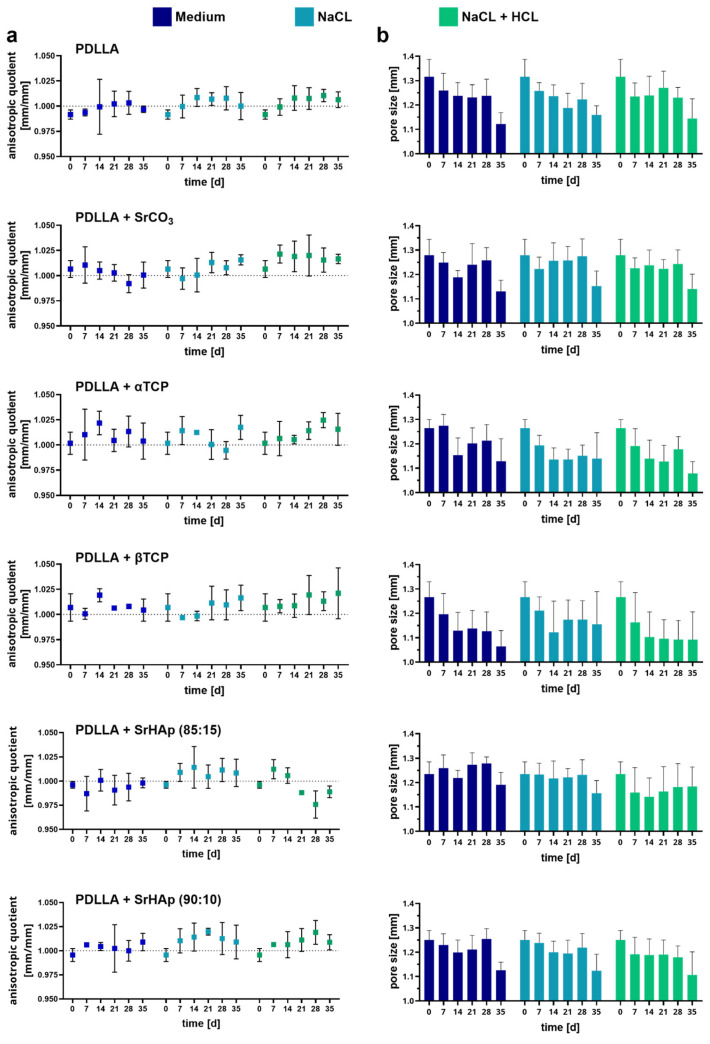
Quantitative analysis of anisotropic swelling of 3D-printed porous scaffolds consisting of PDLLA blended with different mineral phases in comparison to pure PDLLA in the 3D-printed degradation model; incubation was carried out in cell culture medium, 0.9% NaCl, or 0.9% NaCl + HCl (pH4.2) at 37 °C: (**a**) anisotropic quotient, reflecting the ratio of the scaffold widths in the open (channel) direction and in the closed direction (perpendicular to the channel) and (**b**) the diagonal pore diameters (n = 3, mean ± SD).

A more in-depth analysis of swelling in the model was carried out by determining the anisotropic quotient—the ratio of the scaffold widths in the open (channel) direction and in the closed direction (perpendicular to the channel)—and the diagonal pore diameters until day 35. The PDLLA + CaCO_3_ group was excluded, as the strong swelling of this blend also led to an upward expansion, preventing precise measurement. In all cases, the anisotropy quotient is close to 1, indicating no or only minimal anisotropic expansion of the scaffolds (Figure 11a). There are no significant differences between the different incubation solutions and analyzed time points. For all variants, a tendency towards reduced pore sizes with increasing incubation time is recognizable; however, neither the differences between the three incubation solutions nor the analyzed time points were significant (Figure 11b).

## 4. Discussion

In the present study, the thermoplastic polymer PDLLA was blended with microparticles consisting of mineral phases with different solubilities and acidity/basicity [19]. As recently reported for PLLA-PGA (poly(L-lactic-co-glycolic) acid) blended with the same mineral phases [7], processing of these blends using the Arburg Plastic Freeforming process was possible with comparable accuracy (Figure A1). Cylindrical model scaffolds of small dimensions (d = 10 mm, h = 5 mm) were used to study the influence of the mineral phases on degradation and cytocompatibility of the PDLLA-based implant materials in vitro. Finally, PDLLA + CaCO_3_ was selected for a pilot in vivo experiment in two minipigs to gain first insights in the performance of patient-specific fabricated implants of dimensions that are clinically relevant for jawbone defects after tumor resection (length × width × height for animal #1: 29 × 19 × 23 mm and animal #2: 29 × 20 × 20 mm).

Polyesters start to degrade by hydrolysis of the ester bonds. Therefore, penetration of water into the material is a crucial factor that is strongly influenced by the material composition. Since water only penetrates into amorphous polymer domains [20], PDLLA degrades faster than PLLA or PDLA, which both possess higher crystallinity [2,5]. Water penetration can also be influenced by blending with inorganic compounds [21]. As degradation progresses, carboxylic acid end groups accumulate in the amorphous phase, which autocatalytically accelerates the hydrolysis; later, short fragments and monomers dissolve in the aqueous environment, leading to a decrease in the pH value [20]. In our study, a decrease in the pH value in the supernatant of pure PDLLA scaffolds was observed after 12 weeks of incubation, indicating the starting point of dissolution of monomers from the matrix. This process continued during further incubation, as indicated by a further drop in pH and a sharp increase in conductivity in the supernatant of the same scaffolds (Figure 4); the latter was caused by dissociation of the monomers into ionic products. The degradation of the polymer was confirmed by DSC (Figure 5), which revealed a lower glass transition temperature (T_g_) after 24 weeks compared to the early phase (up to week 4) of incubation. The reduced T_g_ can be attributed to a higher proportion of crystalline domains due to the degradation of the amorphous domains. It was also confirmed by measurement of the viscosity (Figure 6), which indicated a continuous length reduction in the polymer chains with incubation time.

The integration of the mineral phases makes the degradation process more complex. That is reflected by changed properties of the scaffolds fabricated from the blends. As expected from previous studies with PLA derivatives [7,8,9,22,23,24], all blends showed a clearly reduced (in the case of the phosphate phases) or nearly no (in the case of the carbonate phases) drop in the pH value in the supernatant (Figure 4). Therefore, a buffer effect of the mineral phases and/or their ionic dissolution products is obvious. Buffering can occur both in the aqueous environment of the scaffolds by ions released from the mineral phases into the supernatant as well as in the local microenvironment of the amorphous domains inside the polymer material, influencing the autocatalytic degradation process [8,16,21].

Dissolution of the mineral particles in the blends is evidenced by the occurrence of pores in the polymer matrix of all blends but not of pure PDLLA (Figure 3). It is further evidenced by conductivity measurement (Figure 4): a nearly constant increase in conductivity of the supernatants of the blend scaffolds over the incubation time of 24 weeks, which clearly differs from the curve of pure PDLLA, indicates a steady release of ions from the mineral phases. Both pore formation and conductivity were highest for PDLLA blended with CaCO_3_ in comparison to the other blends. The reason for this observation might be a faster dissolution of CaCO_3_ compared to the other mineral phases and thus the release of calcium and carbonate ions, as suggested previously for the composite of PLLA-PGA and CaCO_3_ [7]. This assumption is supported by the solubility product constant (Ksp), which is highest for CaCO_3_ (3.8 × 10^−9^) compared to those of the SrCO_3_ (1.1 × 10^−10^) and calcium phosphate phases (Ca_3_(PO_4_)_2_: 1.0 × 10^−25^).

It can be speculated that the pore formation increased the specific surface area and thus facilitated water penetration into the polymeric matrix. Moreover, the integrated mineral phases might have increased the hydrophilicity of the polymer blends in comparison to pure PDLLA, as demonstrated by Bayart and coworkers for PLA + HAp blends [25] and by Li and Chang for PDLLA + wollastonite composites [22]; that might favor water penetration as well. We have monitored the dimensional change in the scaffolds over the degradation period of 24 weeks, as swelling is an indicator of water uptake and thus reflects the initial stage of degradation [19]. In line with the aforementioned expectations, swelling was observed to be significantly increased for all blends from week 4 onwards in comparison to pure PDLLA, which showed only a marginal increase in scaffold dimensions at week 24 (Figure 2). The strongest swelling, indicating the highest water uptake, was observed for PDLLA + CaCO_3_; this observation confirms findings of our previous study investigating PLLA-PGA-based blends [7] and other studies comparing composites of PLGA with CaCO_3_ and various phosphate phases [8,19]. Again, this finding is consistent with the high solubility constant of CaCO_3_ (3.8 × 10^−9^).

The increased water uptake observed for the blend groups can be expected to accelerate the hydrolytic degradation of PDLLA. On the other hand, the buffer effect is expected to prevent autocatalysis and thus decrease the degradation of PDLLA. Also, the formation of the pores might reduce local autocatalysis, since the accumulation of acidic degradation products in the interior of the implant might be prevented [9]. The data of the DSC analyses clearly indicate a diminished polymer degradation of all blends, since the glass transition temperature after 24 weeks of incubation was clearly higher in the case of the blends compared to the pure PDLLA group (Figure 5). A higher T_g_ means that more energy is needed to achieve glass transition, and thus that the proportion of amorphous domains is higher in the blends as a result of less degradation. The different degradation behavior of the blends is also reflected by the absence of recrystallization (indicated by the absence of crystallization and melt peaks at temperatures higher than T_g_) during degradation, which might also be a hint toward decelerated degradation. Also, the measurement of viscosity indicated a retarded polymer degradation, since the drop in viscosity from week 0 to 24 is clearly lower in the case of the blends as compared to pure PDLLA (Figure 6). Similarly, a retarded degradation was reported for PDLLA + 60 wt% CaCO_3_ (coral) [26] and PLGA + 30% CaCO_3_ blends [19]. However, there are also contradictory reports in the literature [9,27,28], and as discussed recently [7], variations in the polymer composition, the amount, size, and shape of the filler particles, the fabrication procedure of the blend specimens, and the aging conditions might be the reason for these differing observations.

The cytocompatibility of PDLLA blended with the different mineral phases in comparison to pure PDLLA was studied by utilizing the human osteosarcoma cell line SAOS-2, which is a standardized cell line often used as a model for osteoblast cells in cytocompatibility analyses [29]. Cytocompatibility was proven for all material groups in the in vitro cell culture experiment (Figure 7 and Figure A2). The sometimes high standard deviations (Figure 7) are most likely a result of the static seeding procedure, which is prone to heterogeneity—a variable proportion of the seeded cells can attach to the well plate (diameter 10.5 mm) instead of the scaffold surface (diameter 10 mm). Dohle et al. recently reported on in vitro investigations of the PDLLA + CaCO_3_ blend using primary osteoblasts, primary endothelial cells, and primary dermal fibroblasts. For all three cell types, they observed high cell viability as well as excellent cell adhesion and expression of their typical markers in contact with the sample surface [30]—these cell responses were comparable to the results obtained for PDLLA + CaCO_3_ and PDLLA in the present study. For 3D-printed scaffolds of PDLLA + β-TCP, excellent cell attachment, viability, proliferation, and osteogenic differentiation of adipose-derived stem cells seeded on these scaffolds were confirmed by Rezai Rad et al. [31]. In addition, in our recent study on PLLA-PGA-based blends, we observed comparable or even increased proliferation and osteogenic differentiation in the presence of the mineral-phase particles in cell culture experiments with SAOS-2 and human dental pulp stem cells [7].

The incorporation of strontium ions into the material by blending PDLLA with SrCO_3_ and SrHAp was undertaken because strontium ions are known to promote the growth and osteogenic differentiation of mesenchymal stromal and osteoblast cells and attenuate osteoclastic resorption, which makes strontium particularly interesting for patients with osteoporosis [32,33,34]. However, as already found previously for the PLLA-PGA blends (PLLA-PGA + SrCO_3_, PLLA-PGA + SrHAp) [7], no positive effect on cell number or ALP activity was observed for the strontium-modified blends. This can most likely be traced back to a very low release of the strontium ions [7].

Among all material variants tested in vitro, PDLLA blended with CaCO_3_ displayed the best buffering effect and the fastest degradation of all blends, properties that might be beneficial for bone regeneration. Thus, scaffolds made of PDLLA + CaCO_3_ were chosen for investigation in a first in vivo trial experiment.

In the current study, the mandibular defect in the minipig jaw was created as a full-thickness, approximately 3 cm segmental defect involving the tooth-bearing section (Figure A2a). Since the periosteum was preserved during surgery, the defect is considered to be non-critical [35]. Nevertheless, this defect model is challenging, since even with careful plastic coverage, an open connection to the oral cavity might occur, allowing for the penetration of bacteria into the defect region and, consequently, the scaffold area. On the other hand, this dynamic process reflects the clinical scenario quite well and might give more realistic results.

The regeneration of such large bone defects in the minipig jaw as a function of periosteum preservation was investigated by Ma et al. [35]. They found that when removing the periosteum, small defects of 1 and 2 cm did not heal during a 12-week observation time. In contrast, when preserving the periosteum—as done in our study—4 cm segmental defects were nearly fully restored after a healing time of 4 weeks, and bone remodeling occurred at 8 and 12 weeks after the operation. To further enhance this bone regeneration and shorten the time until the defect is fully bridged, implants are used as a guide track for the bone being reconstructed. Since degradation of the implant material is associated with a decrease in mechanical properties, there is an ideal volumetric balance between implant material degradation and the formation of new bone to transfer the load from the implant to the bone. For the bone defect model used in our study, and based on the observations by Ma et al., we assumed that an implant degradation time of about 3–6 months would be optimal. As observed by a decreasing pH value, the PLLA-PGA investigated in our former study [7] showed quite a fast degradation compared to PDLLA (Figure A6). Since acidic conditions in the surrounding area or tissue—as can occur in the case of infections—can accelerate the degradation, we decided to use PDLLA instead of PLLA-PGA as the polymer base for the first in vivo trial. For other kinds of bone defects, or if the periosteum cannot be preserved, longer implant degradation times might be needed. For these cases, PLLA would be superior to PDLLA: when implanted in the dorsal muscle of rats, PDLLA was totally resorbed after 72 weeks [36], whereas PLLA implanted into the distal femur or proximal tibia of sheep was still present as fragments after 3 years [37].

**Figure A4:**
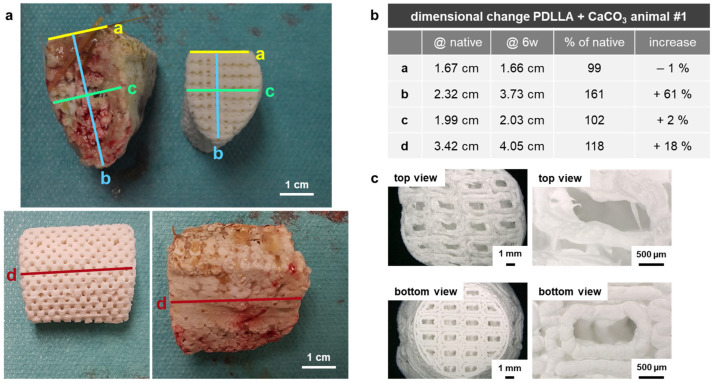
Swelling of PDLLA + CaCO_3_ in vivo vs. in vitro. Comparison of swelling between native and explanted scaffold of animal #1 (**a**,**b**) and swollen PDLLA + CaCO_3_ scaffold at the top and at the bottom after 4 weeks of incubation in vitro (**c**). In contrast to the scaffold explanted from the animal, pores of the in vitro scaffold remained open at both sides due to homogeneous swelling.

To account for swelling that was observed in vitro, implants for the minipigs were not produced as a “perfect fit” into the defect. New bone formation mainly starts from the osteotomy borders, which is why it is important to have close contact between the bone and the implant. Thus, in the bone defect model used in our study, the implant was only reduced in height (Figure A2a, animal #1: height reduced by 10 mm, animal #2: height reduced by 17 mm) to ensure proper contact with the resection/osteotomy borders and because swelling in the cranial direction is most critical due to the sensitive sutured oral mucosa. In addition to fixation between the osteotomy borders, the implant was fixed to the osteosynthesis plate using small screws made of PDLLA. Despite the implant height reduction, in both minipigs, strong swelling of the scaffolds occurred, similarly to the in vitro observations that led to intraoral dehiscences 5 weeks postoperatively. Due to the fact that the scaffold is fixed between the osteotomy/resection borders and the reconstruction plate, swelling in vivo is nearly only possible in the cranial (=scaffold height) and—due to the constant pressure of the tongue—only to a slight extent, in the lingual (=scaffold diameter) direction. Therefore, measurement of the swollen scaffold explanted from animal #1 showed nearly no change in scaffold length and diameter, whereas scaffold height was increased by approximately 61% as compared to the native scaffold (Figure 8c and Figure A4). In contrast to the scaffolds explanted from the animals, pores of PDLLA + CaCO_3_ scaffolds incubated for 4 weeks in vitro remained open due to not limited, homogeneous swelling (Figure A4a,c).

**Figure A5:**
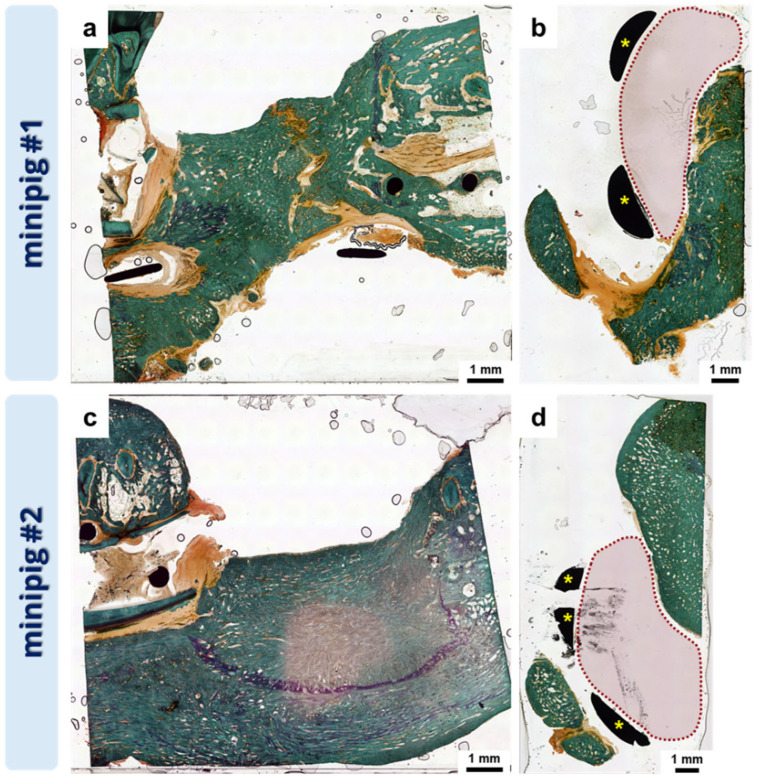
Histological evaluation of the newly formed bone within the defect area 6 month postoperation in sagittal (**a**,**c**) and coronar (**b**,**d**) direction (Goldner staining). The red dotted area in (**b**,**d**) depicts the area between the osteosynthesis plate and the newly formed bone where the implant was originally placed (yellow asterisks = osteosynthesis plate).

**Figure A6:**
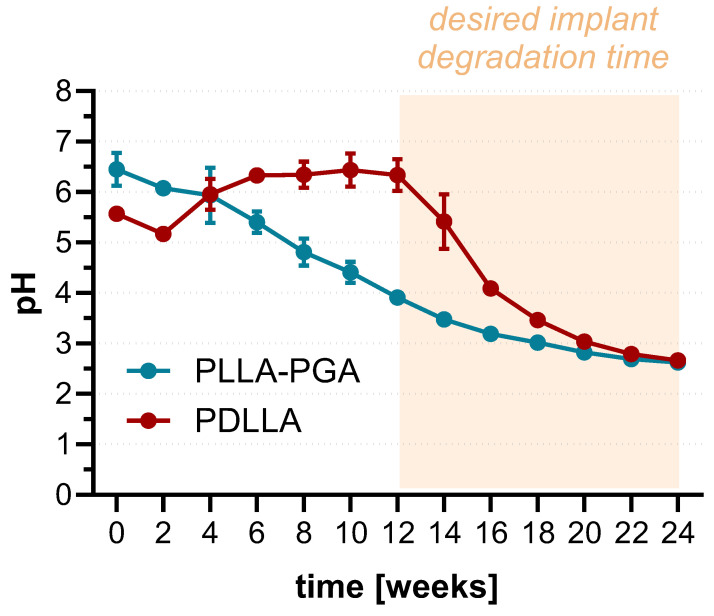
In vitro degradation of PDLLA vs. PDLLA-PGA in terms of pH development. Measurement of pH value in the supernatant of 3D-printed porous scaffolds consisting of PLLA-PGA or PDLLA during aging in water over 24 weeks (n = 3, mean ± SD).

When preserving the periosteum, new bone formation starts from the osteotomy/resection borders as well as from the periosteum [38]. In line with this, bone formation in our study started from the osteotomy borders along the reconstruction plate and around the implant (Figure 9 and Figure A5). No signs of new bone growth from the periosteum into or within the scaffold itself were visible. Moreover, although scaffolds were removed 6 (minipig #1) and 8 weeks (minipig #2) after implantation, respectively, no new bone formation occurred where the scaffolds were originally placed (Figure A5). This indicates an early hampering effect of the scaffolds themselves on bone regeneration. Kauffmann and coworkers observed a similar effect for particles made of PDLLA/CaCO_3_ that were implanted into 3-month-old, not already healed bone defects in the alveolar crest of the maxillae of minipigs [39]. After 4 weeks of healing, PDLLA/CaCO_3_-treated animals showed less newly formed bone compared to SHAM-operated animals, with no signs of the induction of bone formation between the PDLLA/CaCO_3_ particles that were furthermore separated from the underlying native bone by a fibrous tissue layer. Also, for other PDLLA devices like foils, screws, or pins, a tendency toward fibrous encapsulation has been described [5]. Since the scaffolds in our study had to be removed from the animals after 6 and 8 weeks, respectively, histological evaluation of the defect area 6 months postoperatively showed only minor signs of fibrous tissue around the newly formed bone (Figure A5a,b), but a clear absence of newly formed bone at the site where the implant was initially placed; regenerated bone was observed only around the implant site (Figure A5b,d).

Another possible reason for the missing new bone formation in the scaffold area might be the degradation of the scaffold and its effect on the surrounding tissue. In vitro PDLLA + CaCO_3_ showed a constant pH buffering effect over 24 weeks, whereas pure PDLLA decreased the pH clearly beginning after 12 weeks. Intraoral dehiscences caused by swelling of the scaffolds allow for bacteria access into the defect/scaffold area, which induces an inflammatory reaction that is associated with a reduction in pH and a recruitment of various cells [40,41]. By changing the microenvironment, scaffold degradation as well as bone regeneration is influenced. Along with the bacterial infection-induced decrease in pH, scaffold degradation and possibly also swelling might have been enhanced, and thus the addition of 15 w% CaCO_3_ to the PDLLA might not be sufficient to buffer acidification, leading to an increased inflammatory reaction in the surrounding tissue, fibrous encapsulation, and therefore hampered new bone formation within or into the scaffold. Additionally, in contrast to other PDLLA-based devices used in dentistry like foils, screws, or pins, in our study, the amount of PDLLA within the scaffolds used for the in vivo testing is much higher (PDLLA pin 2.1 × 7 mm ≈ 0.024 cm^3^, scaffold for in vivo in our study ≈6.8 cm^3^). A higher amount of PDLLA, in turn, generates a higher amount of acidic degradation products. Especially in areas like the lower jaw, where there is less soft tissue or muscle as compared to, e.g., the femur, these degradation products might not be balanced/metabolized adequately by the tissue, leading to different results when implanting the same scaffold in different anatomical regions.

## 5. Conclusions

For the poly(lactic acid) isomer PDLLA, the in vitro experiments have proven that blending with mineral phases in general is a promising approach to buffer acidic degradation products and to modulate degradation while maintaining cytocompatibility. The results obtained in the in vivo experiment revealed that PDLLA blended with CaCO_3_ is not suitable for the restoration of bone defects with dimensions exceeding these of the already clinically established foils, pins, and screws. On the other hand, PDLLA blended with the other mineral phases studied herein might be promising and will be investigated in this in vivo model in the future. Adaptation of existing in vitro models to better mimic the complex in vivo environment in clinical situations is suggested. That can be realized, e.g., by evaluating scaffolds with a realistic shape and size in defect-simulating 3D models to predict the behavior (swelling, stability, degradation) of newly developed implant materials in vivo more precisely.

## Figures and Tables

**Table 1:** Upper surface area [mm^2^] measured at different time points of incubation in cell culture medium at 37 °C (n = 3; mean ± SD).

	PDLLA	PDLLA + CaCO_3_	PDLLA + SrHAp (90:10)
0 d	79.1 ± 1.9	80.3 ± 0.4	79.8 ± 0.6
7 d	81.1 ± 1.1	90.7 ± 3.0	82.6 ± 0.2
14 d	81.4 ± 0.1	95.4 ± 4.3	83.5 ± 0.6
21 d	79.9 ± 1.0	100.4 ± 5.8	82.5 ± 0.6
28 d	79.1 ± 0.8	102.3 ± 7.3	82.6 ± 0.4
35 d	78.6 ± 0.9	109.8 ± 8.7	82.9 ± 0.7
42 d	78.7 ± 1.0	111.9 ± 10.2	83.7 ± 0.9
49 d	81.8 ± 1.3	116.4 ± 10.6	87.5 ± 0.7
56 d	82.3 ± 1.4	118.4 ± 11.6	88.8 ± 0.7
63 d	82.6 ± 1.4	118.7 ± 11.7	88.8 ± 0.8

## Data Availability

The raw data supporting the conclusions of this article will be made available by the authors on request.

## Appendix A

**Table A1:** In vitro degradation model for prediction of swelling. Upper surface area [mm^2^] measured at different time points of incubation in 0.9% NaCl at 37 °C (n = 3; mean ± SD).

	PDLLA	PDLLA + CaCO_3_	PDLLA + SrHAp (90:10)
0 d	79.2 ± 0.9	78.3 ± 1.6	79.5 ± 0.9
7 d	80.8 ± 0.3	91.3 ± 0.4	83.2 ± 0.9
14 d	81.8 ± 0.3	96.4 ± 2.6	83.7 ± 0.9
21 d	80.0 ± 0.3	100.3 ± 2.6	82.6 ± 0.9
28 d	80.0 ± 0.3	105.2 ± 3.1	83.3 ± 1.4
35 d	79.6 ± 0.5	109.4 ± 5.1	83.4 ± 1.5
42 d	79.5 ± 0.6	109.3 ± 2.7	84.3 ± 1.3
49 d	82.2 ± 0.3	115.9 ± 2.8	87.1 ± 1.3
56 d	82.9 ± 0.5	118.9 ± 3.5	88.7 ± 1.0
63 d	82.5 ± 0.1	120.2 ± 3.8	89.7 ± 0.4

**Table A2:** In vitro degradation model for prediction of swelling. Upper surface area [mm^2^] measured at different time points of incubation in 0.9% NaCl + HCl (pH 4.2) at 37 °C (n = 3; mean ± SD).

	PDLLA	PDLLA + CaCO_3_	PDLLA + SrHAp (90:10)
0 d	78.9 ± 0.4	79.1 ± 1.0	80.6 ± 0.2
7 d	81.1 ± 1.0	89.6 ± 2.3	82.4 ± 0.7
14 d	80.9 ± 0.8	94.8 ± 2.5	83.0 ± 0.6
21 d	79.9 ± 0.7	99.4 ± 2.5	82.5 ± 1.2
28 d	79.9 ± 0.3	107.0 ± 4.1	83.3 ± 1.1
35 d	78.8 ± 0.7	105.8 ± 3.9	83.6 ± 1.2
42 d	78.5 ± 0.3	107.7 ± 6.1	83.8 ± 1.5
49 d	81.8 ± 0.8	113.6 ± 6.9	86.8 ± 1.6
56 d	82.6 ± 0.6	117.1 ± 10.9	88.9 ± 1.1
63 d	82.1 ± 0.7	119.2 ± 11.0	89.7 ± 1.8
